# Investigation of haematological, inflammatory parameters and the incidence of alloimmunization in multi-transfused sickle cell diseased patients

**DOI:** 10.1016/j.htct.2025.103937

**Published:** 2025-07-09

**Authors:** Florence Ifechukwude Aboderin, Taofeeq Oduola, Glenda Mary Davison, Oluwafemi Omoniyi Oguntibeju

**Affiliations:** a Department of Biomedical Sciences, Cape Peninsula University of Technology, Faculty of Health and Wellness Sciences, Bellville 7535, South Africa; b Department of Medical Laboratory Science, Faculty of Allied Health Sciences Ila-Orangun, Bellville 7535, South Africa; c SAMRC/CPUT Cardiometabolic Health Research Unit, Department of Biomedical Sciences, Cape Peninsula University of Technology, Bellville 7535, South Africa

**Keywords:** Haematological, Inflammatory parameters, Alloimmunization, Multi transfused, Sickle cell disease

## Abstract

**Introduction:**

Sickle cell disease is a haemoglobinopathy caused by an aberrant mutation of the beta chain with the amino acid valine replacing glutamic acid at the 6th position. Patients with sickle cell disease suffer from complications including chronic inflammation and the development of allogeneic antibodies due to multiple blood transfusions. This study investigated the association between haematological, inflammatory markers and alloimmunization in multi-transfused patients with sickle cell disease.

**Methods:**

This was a cross-sectional study, that enrolled 100 participants; 50 young adults (18–48 years) with homozygous sickle cell disease (Sickle cell Group) from the Obafemi University Health Centre in Nigeria, and 50 age and sex matched individuals who did not have the disease (Control group) but who had also received blood transfusions. Complete blood counts and differentials were processed on an auto-analyser (SFRI H18 Light, France). Red cell antigen identification used the saline and anti-human globin method while the abnormal haemoglobinopathy was evaluated using electrophoresis. ABO and Rhesus blood groups were analysed using a direct method on tile, and the determination of inflammatory markers including C-reactive protein, tumour necrosis factor-alpha, interleukin-6, and interleukin-1β was by the enzyme-linked immunosorbent assay technique. The data were statistically analysed using SPSS version 24.0 and GraphPad Prism. Additionally, the student *t*-test and Chi-square test were employed as appropriate. Data were presented as mean ± standard deviation, with a *p*-value <0.05 considered statistically significant**.**

**Result:**

As expected, the Sickle Cell group had an increased rate of alloimmunisation and significantly reduced haemoglobin and red cell parameters except for the mean cell volume. Although both groups had platelet counts within the reference range the Sickle Cell group had significantly higher counts than the Control group. The Sickle Cell group displayed evidence of inflammation with significantly increased levels (*p*-value = 0.001) of C-reactive protein and tumour necrosis factor-alpha. This was supported by higher white cell counts and neutrophilia. The majority of the antibodies detected in sickle cell disease were anti-Kell, Jka and Fya while the controls showed a higher prevalence of anti-M and Kell antibodies. Despite the elevated inflammatory markers, no significant correlation was observed between these and the rate of alloimmunization.

**Conclusion:**

In this study, the Sickle Cell group had an elevated rate of alloimmunization with higher levels of anti-kell, Jka and Fya as well as inflammatory markers. However, despite these findings, no significant correlation between inflammatory markers and alloimmunization could be detected. This suggests that elevated alloimmunization rates are multifactorial and involve other processes which require further investigation.

## Introduction

Sickle cell disease (SCD) is a genetic disorder characterized by haemoglobin S (Hb S) resulting from the inheritance of an abnormal beta-globin chain gene from one or both parents. Globally, 50 million people are affected, with Africa experiencing from 50–90 % of childhood mortality.^1^ In Ghana, screening of newborns between 1995 and 2004 recruited 177,283 newborns with 3346 having SCD. Annually, SCD makes up about 2 % of 5 million total births per year.^2^ Screening is to ensure that SCD in children is captured early enough for medical treatment. Meanwhile, in Nigeria, it has been estimated that 24 % of the population has the sickle cell trait (SCT), with an estimated 150,000 babies born with SCD annually. SCD makes up 3 % of the total births while the annual infant mortality rate is approximately 100,000.^3^

In Africa, blood transfusion remains the most common form of therapy for SCD effectively reducing complications including vaso-occlusive crises, acute pain, and chest pain syndrome by increasing the oxygen-carrying capacity of blood.^4^ However, there are risks associated with blood transfusions such as blood-borne diseases, and allergic and haemolytic reactions. Chronically transfused patients suffer from iron overload and alloimmunization which is particularly prominent in those suffering from SCD. The production of alloantibodies can affect up to one-third of the SCD population potentially resulting in delayed haemolytic transfusion reactions (DHTR).^5^ Identifying suitable and compatible blood for a patient with multiple alloantibodies has therefore become a challenge.^6^

The presence of delayed reactions and the development of antibodies in multi-transfused patients has been implicated in various pathological conditions. It has been proposed that one of the causes of the increased alloimmunization rate in individuals with SCD is chronic inflammation.^7^

Inflammation arises from an abnormal activation of innate immune responses which can be initiated by the process of haemolysis. This commences chronically in SCD when red blood cells are damaged, releasing various molecules into the peripheral blood, including Hb S and heme (iron compound). It was recently discovered that free heme increases in both SCD and beta thalassaemia, however, the inflammation in SCD is triggered by circulating abnormal haemoglobin.^8^ The abnormal Hb S binds to Toll-like receptor 4 (TLR4: also known as CD284) expressed on monocytes, a key activator of the innate immune response.^9^^,^^10^ The resulting inflammation has been correlated with mortality and therefore it has been hypothesized that elevated levels of pro-inflammatory markers could predict the buildup of allogeneic antibodies.

It has been observed in a murine model that inflammation plays an important role in RBC alloimmunization.^11^ This work supported the theory that SCD is characterized by chronic inflammation^12^ and has led to the hypothesis that inflammation plays a role in the increased rate of alloimmunisation observed in these patients despite little published data. This hypothesis appears reasonable because inflammatory signals activate the immune response and advance the recognition of foreign antigens. The release of cytokines, such as interleukin-6 (IL-6), interleukin-1 (IL-1) and tumour necrosis factor-alpha (TNF-α) as well as the activation of antigen-presenting cells and tissue damage may lead to initiation of alloimmunization when foreign antigens are introduced during transplantation and transfusion.

Alloimmunization poses a complex challenge with the risk increasing after every additional blood transfusion.^13^ A study carried out in the United States reported that 50 % of all immunized subjects had multiple antibodies.^14^, ^15^, ^16^ Over time many of these became undetectable, potentially challenging future transfusion and putting the patient at risk of a DHTR.^17^ The most common red cell antigens involved are the Rh, Kell, Kid, Duffy, Lewis, and MNS blood group systems.^18^^,^^19^ Other factors include the recipient's age and sex, number and frequency of transfusions, history of pregnancy, recipient clinical diagnosis and treatment, ethnic differences between recipient and donors and genetic factors related to antigenic responses.^16^

The overall incidence of post-transfusion alloimmunization in Nigeria varies from 18.7 %^20^ to lower rates of 8.8 %^21^ depending on the region where the study took place.

With this background, it has been hypothesized that those with elevated levels of inflammatory markers have a higher risk of alloimmunization and delayed transfusion reactions. Therefore, this study aimed to investigate the association between the increased incidence of alloimmunization in multi-transfused individuals with SCD and chronic inflammatory markers. This study focused on a cohort of individuals with SCD diagnosed at the haematology department of an academic hospital in Nigeria.

## Research design and methodology

This was a cross-sectional study conducted at the Department of Haematology and Immunology, Obafemi Awolowo University Teaching Hospital, Nigeria. The complete blood count (CBC), differential count, inflammatory markers, and blood group antigen profiles of patients with SCD (SCD group) who received two or more doses of blood were compared to a group of individuals without the disease (Control group) but who also received blood transfusions.

### Inclusion and exclusion criteria

All patients who were diagnosed with SCD who had received at least two blood transfusions were included in the SCD group. The Control group was made up of age and sex-matched individuals who did not have SCD who had received at least two blood transfusions. Individuals who were experiencing a sickle cell crisis were excluded.

### Ethical consideration

The study received ethical clearance from both the Human Research Ethics Committee, Faculty of Health and Wellness Sciences, Cape Peninsula University of Technology, (CPUT/HWS-REC2021 renewal) Bellville, South Africa and the Research and Ethics Committee of Obafemi Awolowo University Health Centre, Ref: (D.MHS/2023) Ile-Ife. Informed written consent was obtained from all participants involved in the research.

### Diagnosis of sickle cell disease

All patients with SCD were diagnosed according to established criteria using traditional haematological parameters including blood smear morphology. Thereafter the diagnosis was confirmed using haemoglobin electrophoresis which was performed on a Helena manual electrophoresis instrument (Helena Biosciences, UK) according to a previously described method.^22^

### Analysis of samples

Six millilitres of blood were collected into ethylenediaminetetraacetic acid (EDTA) and serum separator tubes. A CBC, haemoglobin electrophoresis, ABO and Rhesus blood group typing and red cell antibody typing of Rh (D, C, E, c, e), Kell (K, k), Duffy (Fya, Fyb) and Kidd (Jka, Jkb) M, N, S, s, PI, Lu^a^, Kp^a^, Le^a^, Le^b^ were investigated in both the SCD and Control groups. The red cell antibodies were interpreted using the ID panel profile. TNF-α, C-reactive protein (CRP), IL-6, and Interleukin-1 beta (IL-1β) were also analysed using enzyme-linked immunosorbent assays (ELISA).

### Complete blood count and blood smear analysis

CBCs were performed using an H18Light auto analyser (SFRI, France) which uses the impedance technique to enumerate blood cells and spectrophotometry to determine haemoglobin levels. Red cell indices were calculated using the red cell count and haemoglobin values. Before analysis, three levels of control were used to ensure the accuracy of the autoanalyzer.

A routine blood smear was stained with Leishman stain for 3–5 min and after washing the slides they were allowed to dry before examining under an x100 objective. A manual differential was performed, and the red cells were examined for the presence of sickling and other red cell abnormalities.

### Haemoglobin electrophoresis

Blood samples were haemolysed using hemolysate and an appropriate volume of Tris buffer (pH 8.4) was added to the electrophoresis chamber. Cellulose acetate paper was soaked for 20–30 min in the buffer, after which the excess was blotted and 0.5–0.6 mL of the specimen was applied. The cellulose acetate paper was placed in the electrophoresis chamber and covered to run at 450 V for 20 min. The cellulose paper was then removed and stained with Ponceau S for three minutes. The results of the abnormal haemoglobins were compared with the relative mobility of control samples.^23^

### Determination of ABO and rhesus blood groups

Biotech’s blood grouping reagents for ABO and Rhesus were used to determine the blood groups. This was achieved by tile grouping and confirmed by tube grouping methods utilizing anti-sera A, B and D for Rhesus, while tube grouping employed pooled A, B and O cells. Equal volumes of each type of cell and antisera were added and mixed and agglutination was observed and interpreted appropriately. Red cell antibody analysis was carried out to determine the presence of clinically significant red cell antibodies such as Kell, Kidd, and Lewis which could cause alloimmunization. This was achieved using the ID panel cells for red cell antibodies (ID Panel cells, product code PR144, NHSBT Reagents) which were processed according to the manufacturer’s instructions (NHS Blood Transplant PR 1444–2020).

### Inflammatory markers

ELISA KITS for TNF-α, CRP, IL-6, and IL-1β (Elabscience Biotechnology, USA) were used for the measurement of inflammatory cytokines. The micro-Elisa plates were pre-coated with antibodies specific for Human TNF-α, CRP, IL-6, and IL-1β. Samples (or standards) were added to the micro-ELISA plate wells and incubated with each specific antibody. Thereafter, a biotinylated detection antibody and avidin-horseradish peroxidase (avidin-HRP) conjugate was added to all the microplate wells. Free components were washed away, and a substrate solution was added. Only those wells that contained human TNF-α, CRP, IL-6, and IL-1β, biotinylated detection antibody and Avidin-HRP conjugate appeared blue and the reaction was terminated by the addition of a stop solution resulting in the colour turning yellow. The optical density (OD) was measured spectrophotometrically at a wavelength of 450 ± 2 mm with the OD values being proportional to the concentrations of the relevant analyte. The concentrations of each analyte were calculated by comparing the OD of all the respective samples to their standard curves.

### Statistical analysis

Data were subjected to statistical analysis using SPSS version 24.0 and the GraphPad Prism version 8 statistical package and relevant statistical values were obtained. Student *t*-test was used and data were presented as means ± standard deviation (SD). The Chi-square test was also employed where appropriate. *P*-values <0.05 were considered statistically significant.

## Results

### Demographics and clinical characteristics

All participants were of African descent and between the ages of 18 and 48 with no significant difference between the mean age of those with (30.66 ± 9.25 years) and without (31.46 ± 9.87 years) SCD. Thirty of the SCD group were females compared to 26 in the Control group. As expected, those suffering from SCD received significantly more transfusions (165 versus 108) compared to the controls (*p*-value <0.001). On average each person in the SCD group received 3.4 transfusions compared to 2.2 in the Control group (*p*-value <0.0001). Nineteen (38 %) of the SCD group experienced a transfusion reaction compared to ten (20 %) of the Control group. Although the number of reactions was higher in those with SCD, there was no significantly difference between groups (*p*-value = 0.2038 - Table 1). The types of transfusion reactions observed were mostly haemolytic reactions and included allergy, coldness, shivering, rash, and itching.

**Table 1:** General characteristics of participants.

	Control*n* = 50 (%)	SCD*n* = 50 (%)	Total*n* = 100 (%)	*P*-value
Age (years)	31.46 ± 9.877	30.66 ± 9.255		0.6769
Gender				
Female	26 (52.00)	30 (60.00)	56 (56.00)	χ^2^ = 0.6494, df =1 *P*-value = 0.4203
Male	24 (48.00)	20 (40.00)	44 (44.00)	
Haemoglobin electrophoresis				
Hb AA	36 (72.00)		36 (36.00)	χ^2^ = 100.00, df =3 *P*-value <0.0001*^+^
Hb AC	5 (10.00)		5 (5.00)	
Hb AS	9 (18.00)		9 (9.00)	
Hb SS		50 (100.00)	50 (50.00)	
The mean number of blood units transfused	2.160 ± 0.5095	3.360 ± 1.045		<0.0001
Mean number of blood transfusion reactions	0.400 ± 0.8571	0.6327 ± 0.9507		0.2038

SCD: sickle cell disease.

### Complete blood count and haematology

As expected, the red cells, haematocrit (Hct), haemoglobin and all red cell indices apart from the mean cell volume were significantly different between the SCD and Control groups. In addition, those with SCD had significantly higher neutrophil counts (*p*-value <0.0001) and a lower lymphocyte count (*p*-value <0.0001). Although both groups had platelet counts within the reference range, those with SCD had a significantly higher platelet count (*p*-value <0.0001 - Table 2).

**Table 2:** Comparing the haematological parameters in sickle cell disease and non-sickle cell anaemia patients.

Parameter	Control group		SCD group		*p*-value
	Mean ± SD	Median	Mean ± SD	Median	
WBC (x10^9^/L)	15.702 ± 40.587	5.550	14.803 ± 6.453	13.450	0.0001^a^
Platelets (x10^9^/L)	182.300 ± 87.196	177.500	289.063 ± 128.976	267.500	0.0001^a^
Hct (%)	31.20 ± 23.13	32.50	23.13 ± 4.741	23.00	0.0001^a^
Hb (g/dL)	10.41 ± 2.852	11.45	7.642 ± 1.482	7.650	0.0001^a^
MCV (FL)	79.72 ± 4.468	80.00	80.75 ± 4.468	80.00	0.3603
MCH (pg)	31.70 ± 11.84	30.00	27.62 ± 6.469	26.00	0.0001^a^
MCHC (g/dL)	31.76 ± 1.364	32.00	31.45 ± 0.9237	31.10	0.0763
Neutrophil (%)	57.74 ± 12.58	59.00	67.60 ± 9.498	70.00	0.0001^a^
Neutrophil (x10^9^/L)	9.883 ± 25.636	3.051	10.206 ± 5.029	9.694	0.0001^a^
Lymphocyte (%)	39.74 ± 12.62	40.00	30.00 ± 9.042	29.00	0.0001^a^
Lymphocyte (x10^9^/L)	4.640 ± 10.137	2.388	4.275 ± 2.025	3.585	0.0001^a^
Monocyte (%)	0.4800 ± 0.8142	0.0000	0.5000 ± 0.8864	0.0000	0.9225
Monocyte (x10^9^/L)	0.628 ± 0.114	0.0000	0.141 ± 0.711	0.0000	0.5315
Eosinophil (%)	1.600 ± 1.917	0.0000	1.600 ± 1.953	1.0000	0.0065^a^
Eosinophil (x10^9^/L)	0.7371 ± 3.494	0.0000	0.2174 ± 0.2651	0.1485	0.0002^a^
Basophil (%)	0.5000 ± 1.111	0.2500	0.4200 ± 0.7025	1.0000	0.6397
Basophil (x10^9^/L)	0.2455 ± 1.135	0.0000	0.0583 ± 0.1015	0.0000	0.3778

SD: standard deviation; WBC: white blood cell; Hb: haemoglobin; Hct: haematocrit; MCV: mean corpuscular volume; MCH: mean corpuscular haemoglobin; MCHC: mean corpuscular haemoglobin concentration.

a Mann-Whitney test: significant difference between the Test and Control groups (*p*-value <0.05).

### Development of alloantibodies

Those with SCD had an increase of alloantibodies with a mean of 0.6 alloantibody reactions compared to 0.4 in the controls (p-value = 0.2038). This was however not statistically significant. The majority of the antibodies in those with SCD were anti-Kell, Jka and Fya whereas the Control group had a higher prevalence of anti-M, as well as anti-Kell antibodies (Table 3).

**Table 3:** Distribution of alloantibodies in the sickle cell and control groups.

Alloantibody	Control*n* (%)	SCD*n* (%)	Total*n* (%)
C	2 (4)	2 (4)	4 (4)
E	5 (5)	2 (4)	7 (7)
Fya	3 (6)	6 (12)	9 (9)
Fyb	1 (2)	2 (4)	3 (3)
Jka	0 (0)	8 (16)	8 (8)
K	9 (18)	9 (18)	18 (18)
Kpa	3 ((6)	4 (8)	7 (7)
Lea	5 (10)	2 (4)	7 (7)
M	12 (24)	5 (10)	17 (17)
N	1 (2)	2 (4)	3 (3)
S	3 (6)	2 (4)	5 (5)
C	5 (5)	2 (4)	7 (7)
Cw	1 (2)	2 (4)	3 (3)
E	0 (0)	2 (4)	2 (2)

SCD: sickle cell disease.

### Inflammation markers

Participants with SCD had significantly elevated levels of CRP and the pro-inflammatory cytokine TNF-α. Table 4 demonstrates the independent-samples *t*-test for the inflammatory parameters of participants by group. The result showed that there was a significant difference in the means between the Control and SCD groups for CRP (t[97] = −3.099; *p*-value = 0.003) and TNF-α (t[97] = −2.449; *p*-value = 0.016). The result also showed that there was no significant difference in the means between the Control and SCD groups for IL-6 (t[97] = 1.380; *p*-value = 0.171) and IL-1β (t[97] = −1.710; *p*-value = 0.090).

**Table 4:** Inflammatory markers in the SCD and control groups.

	GroupControl	SCD					
	M	SD	M	SD	t	df	*p*-value
CRP	1.2	2.78	3.9	5.62	−3.099	97	0.003^a^
IL-6	84.3	101.96	58.1	86.10	1.380	97	0.171
TNF-α	2.8	5.76	8.1	14.31	−2.449	97	0.016^a^
IL-1β	2.5	.38	4.1	6.73	−1.710	97	0.090

M: mean; SD: standard deviation; df: degree of freedom; CRP: C-reactive protein; IL-6: interleukin-6; TNF-α: tumour necrosis factor-alpha; IL-1: interleukin-1.

a Significant differences between the groups.

## Correlation between inflammation markers and alloimmunization

Correlation analysis showed weak positive correlations between alloimmunization and two of the pro-inflammatory markers, CRP, and TNF-α. In contrast, very weak negative correlations between alloimmunization and two other inflammatory markers, IL-1β and IL6, were observed. These however were not statistically significant (Table 5).

**Table 5:** Correlation between inflammatory markers and alloimmunization.

Inflammatory marker	Pearson’s correlation	*p*-value
CRP	0.1127	0.2665
IL6	−0.08318	0.4130
IL-1β	−0.02402	0.8135
TNF-α	0.1179	0.2452
Neutrophil	−0.02975	0.7700
WBC	−0.03534	0.7284

CRP: C-reactive protein; IL-6: interleukin- 6; IL-1 β: interleukin 1beta; TNF-α: tumour necrosis factor-alpha; WBC: white blood cell.

## Discussion

This cross-sectional study aimed to explain the complex relationship between inflammation and alloimmunization in individuals with SCD. It highlights the increased transfusion frequency and transfusion reactions in these patients. The participants with SCD developed allogeneic antibodies which were mostly anti-Kell, Jka and Fya while the non-SCD group developed anti-Kell and anti-M antibodies. Despite the individuals with SCD having significantly elevated levels of pro-inflammatory markers (TNF-α and CRP), no significant correlation was detected with the rate of alloimmunization. This suggests that while inflammation is prevalent in patients with SCD, it does not directly predict alloimmunization.^24^

These findings align with previous research by Kangiwa et al.^20^ which reported similar alloimmunization patterns but at higher rates. Differences in sample size, subject age, regional practices, and transfusion protocols likely contribute to the variance in alloimmunization rates. This study focused on young adults in the Western region of Nigeria, while Kangiwa et al.^20^ included both adults and children in the Eastern region, indicating possible regional differences in antigen prevalence and healthcare practices.

Other studies^25^^,^^26^, ^27^, ^28^ corroborate these findings, highlighting the clinically significant increases in Rhesus and Kell antibodies. The alloimmunization rate in Nigeria is higher compared to other African countries like Ghana^29^ Uganda^30^ and Tanzania.^17^

These differences might be explained by variations in the genetic backgrounds between populations which can influence immune responses. For instance, the prevalence of certain antigens like Kell, Jka, and Fya might vary across different ethnic groups, affecting the likelihood of developing corresponding antibodies. Likewise, variations in blood transfusion protocols and donor screening processes between different regions and hospitals can impact alloimmunization rates and the types of antibodies formed.

At our institution, blood units are prophylactically phenotype-matched between the recipient and the donor using ABO and Rhesus grouping and when compatible given to the recipient without necessarily carrying out antigen testing. However, in this current study, an extended antigen testing panel was performed.

The immunogenicity of antigens may also significantly influence the likelihood of antibody development. Antigens like Kell, Jka, and Fya, which were elevated in the SCD group, are known to be highly immunogenic, leading to a higher prevalence of corresponding antibodies in transfused patients. The Duffy antigen, specifically Fya, is particularly immunogenic and prevalent in African populations, making it a common target for alloimmunization in individuals with SCD.^31^^,^^32^

In this current study, individuals with SCD had significantly higher levels of the proinflammatory proteins TNF-α and CRP while IL-6 and IL1 levels were similar to the Control group**.** The increase in inflammatory proteins has been reported by previous studies^33^ and it has been proposed that these proteins could be used as clinical biomarkers. For example, CRP has been associated with acute chest pain (ACP) and vaso-occlusive crises.^34^ This has been confirmed by others whose findings were consistent with this study.^35^^,^^36^

TNF-α is a cytokine with several properties including the activation of endothelial cells and leucocytes. The action of macrophages and the chemotaxis of inflammatory cells has been implicated in the pathogenesis of various acute and chronic states such as sepsis, chronic infections, and inflammatory conditions. TNF-α plays an essential role in the synthesis of protein and the expression of adhesion molecules in vascular endothelial cells.^37^^,^^38^ In SCD, this cytokine has been proposed as a risk factor for the occurrence of painful crises, as well as being involved in the occlusion of the microcirculation.^39^^,^^40^

High white blood cell counts and neutrophils are also associated with inflammation and during infections, neutrophils are the first cells to respond. Activated neutrophils release enzymes, such as reactive oxygen species, proteases and myeloperoxidase, which combat foreign organisms at the infection site.^41^ These enzymes are also involved in several inflammatory processes.^41^ When adhesion takes place, chemokines and cytokines are produced which go on to stimulate dendritic cells resulting in the presentation of antigens to memory CD4-positive T cells as well as to naïve CD8-positive T cells which consequently leads to the activation of the adaptive immune response.^42^

Several other studies have reported elevated platelet counts in individuals with SCD.^43^, ^44^, ^45^ Increased platelets in this study could contribute to the chronic and acute complications of SCD by promoting molecular and diverse cellular events within the microcirculation that eventually lead to vaso-occlusive and vascular injury.

Despite the increase in inflammatory markers, no significant correlation between the rate of alloimmunization and any of the inflammatory markers could be detected, which was similar to a previous study by Tatari-Calderone et al.^24^ Their study, conducted in Washington, DC, USA, involved 83 children with SCD who received multiple red blood cell transfusions for both the prevention and treatment of disease-related complications. The levels of cytokines were correlated with the development of anti-RBC antibodies within the seven-year period post-recruitment and demonstrated that twelve subjects had significantly elevated levels of all cytokines, both pro-inflammatory and anti-inflammatory. Interestingly, higher levels of cytokines were also found in the patients without anti-RBC allo- or auto-antibodies. Therefore, it was concluded that high cytokine levels were not indicative of alloantibody development and that the increased concentration of multiple cytokines is not a biomarker of either the presence of or susceptibility to the development of RBC alloimmunization.

Several other studies have reported increased inflammation and innate immune activation in individuals with SCD^46^, ^47^, ^48^ however, the pattern of cytokine expression varies.^49^^,^^50^ High plasma levels of TNF-α have been reported while others have suggested that reduced production of IFN-γ was the first evidence of the onset of DHTR in individuals with SCD.^51^

Researchers have shown that various factors, aside from inflammation can influence the development of alloimmunization in SCD. These factors include iron overload, haemolysis, delayed haemolytic transfusion reactions, pregnancy, haemolytic disease of the newborn, infection, genetic factors, the antigenic immunogenicity of RBCs, recipient exposure to foreign donor antigens, the immunological status of the recipient, age at first transfusion, and the duration of transfusions. A further factor which could play a role is differences in the human leukocyte antigen alleles. These findings all warrant future research.^52^^,^^53^

## Limitations

This study was conducted in only one region of Nigeria and therefore the results cannot be applied to the general Nigerian population or other countries within Africa. Regional genetic variations, environmental factors, and healthcare practices can influence the results, thereby limiting the broader applicability of the conclusions. A further limitation was the relatively low number of participants. A larger sample size would have provided more robust data and enhanced the statistical power of the study, allowing for more definitive conclusions. Moreover, only four inflammatory markers were analysed which may have overlooked other relevant biomarkers which could provide additional insights into the conditions being studied.

## Conclusion

Despite these limitations, the results indicate that SCD individuals in this region of Nigeria have high rates of alloimmunization and elevated levels of anti-Kell, Jka, and Fya antibodies, along with inflammatory markers, TNF-α and CRP, compared to those without SCD. However, no significant correlation was found between the inflammatory markers and alloimmunization. This study underscores the multifactorial nature of alloimmunization in SCD and the importance of considering genetic, regional, and procedural factors to optimize transfusion practices. Further research is needed to explore these differences and develop strategies to reduce alloimmunization risks in individuals with SCD.

## Conflicts of interest

The author declares no conflicts of interest
